# Classification of rheumatoid arthritis status with candidate gene and genome-wide single-nucleotide polymorphisms using random forests

**DOI:** 10.1186/1753-6561-1-s1-s62

**Published:** 2007-12-18

**Authors:** Yan V Sun, Zhaohui Cai, Kaushal Desai, Rachael Lawrance, Richard Leff, Ansar Jawaid, Sharon LR Kardia, Huiying Yang

**Affiliations:** 1 Department of Epidemiology, School of Public Health, University of Michigan, 611 Church Street #244, Ann Arbor, Michigan 48104, USA; 2 AstraZeneca Pharmaceuticals, 1800 Concord Place, FOC W1-462, Wilmington, Delaware 19850, USA; 3 AstraZeneca Pharmaceuticals, Mereside, Alderley Park, Macclesfield, Cheshire, SK10 4TG, UK

## Abstract

Using the North American Rheumatoid Arthritis Consortium (NARAC) candidate gene and genome-wide single-nucleotide polymorphism (SNP) data sets, we applied regression methods and tree-based random forests to identify genetic associations with rheumatoid arthritis (RA) and to predict RA disease status. Several genes were consistently identified as weakly associated with RA without a significant interaction or combinatorial effect with other candidate genes. Using random forests, the tested candidate gene SNPs were not sufficient to predict RA patients and normal subjects with high accuracy. However, using the top 500 SNPs, ranked by the importance score, from the genome-wide linkage panel of 5742 SNPs, we were able to accurately predict RA patients and normal subjects with sensitivity of approximately 90% and specificity of approximately 80%, which was confirmed by five-fold cross-validation. However, in a complete training-testing framework, replication of genetic predictors was less satisfactory; thus, further evaluation of existing methodology and development of new methods are warranted.

## Background

Rheumatoid arthritis (RA) is an autoimmune disease causing inflammation and soft-tissue swelling of mainly diarthrodial joints. The disease can lead to considerable loss of mobility due to pain and joint destruction. Over the past decade, an improved understanding of the pathophysiology of the disease has had a big impact on RA therapy, mainly through the use of so-called disease-modifying antirheumatic drugs [1].

Complex human diseases such as RA have complicated genetic architectures [2]. Single-gene association studies indicate that each genetic predictor alone has very weak power to predict the disease status. However, the high heritability identified in many human diseases indicates that the overall genetic contribution to risk is substantial. One alternative to traditional modeling is to create an ensemble of models that capture the inherent heterogeneity and interactions that underlie the complex genetic architecture of common diseases. For example, random forests algorithms combine many decision trees, each of which is a weak prediction model, by randomly selecting the sample and predictors for building each tree to obtain an improved overall prediction [3]. The random forests (RFs) method is capable of handling high-dimensional data such as genome-wide single nucleotide polymorphism (SNP) genotypic data and is known to be resistant to uninformative predictors.

We explored the predictive ability of RFs analysis to identify genetic associations with RA and their interactions with other genetic loci in the North American Rheumatoid Arthritis Consortium (NARAC) candidate gene and genome-wide linkage data sets. Furthermore, we performed cluster analysis using the phenotypic data to identify clinically distinct subgroups of RA patients, which may represent a pathophysiologically heterogeneous patient population. Subsequently, such phenotypic subgroups were used to identify additional susceptibility genes.

## Methods

### Data

The phenotype (NAPHENO) and candidate gene (CANDGENE.DAT) data were provided by the North American Rheumatoid Arthritis Consortium [4] and consist of 839 RA cases from sib-pair families and 855 unrelated controls. SNPs with missing data of 15% or greater were excluded from the analysis, resulting in a total of 17 SNPs from 13 genes (*PTPN22*, *CTLA4*, *HAVCR1*, *IBD5*, *SLC22A4*, *IL3*, *IL4*, *SUMO4*, *MAP3K71P2*, *DLG5*, *CARD15*, *RUNX1*, and *MIF*) being considered. The missing genotypes were estimated using the proximity calculated by RFs on the training subjects [3].

In this study, we generated two replicate data sets in which the subjects were independent within samples and were dependent across samples. This approach ignores dependencies between the data sets that in general lead to inflation of type I error in the replication set. However, we feel that this approach is justified to simplify the confirmatory analyses using the available data. The data set was split into two replication data sets by randomly selecting a case from each affected sib pair for one of the data sets and the remaining case contributing to the second data set, with the controls almost evenly divided into the two data sets. Data set 1 includes 398 cases and 427 controls, while data set 2 includes 387 cases and 428 controls. It should be noted that due to the relatedness between the replication and the analysis data sets, the confirmation from the second data set should be interpreted with caution.

The data from the genome-wide linkage panel (a total of 5742 SNPs, excluding chromosome Y SNPs) were available for 1998 individuals from families with RA patient (NAILL01–NAILL25). For families with two or more siblings, one was randomly selected from each family for data set 1, and the second subject was then randomly selected from the remaining of samples for data set 2. The singletons were randomly divided into the two data sets. Each of the data sets consists of 740 unrelated subjects. The analysis of the replication data set should be considered exploratory.

The RF method is not particularly efficient at imputing missing data for large number of SNPs. Therefore, we applied an extension of the expectation-maximization (EM) algorithm implemented in HelixTree (Golden Helix Inc., Bozeman, MT) to impute the missing genotypes for the genome-wide linkage data set. In a simulation study, this method achieved outstanding performance of imputation accuracy above 95% with missing rate ranging from 1% to 10% (Sun et al., unpublished data).

### Association analysis

The association between each candidate gene SNP and RA status was tested using all samples. Two methods were used for the candidate gene data set to adjust for the degree of relatedness among the affected siblings, a generalized estimating equations (GEE) method with likelihood ratio statistics (SAS 8.2, SAS Institute Inc., Cary, NC, USA, 1999) and Risch and Teng's statistic [5]. Results from the Risch and Teng's statistic and GEE likelihood ratio were consistent so that only results from the GEE analysis are reported. SNPs with an association with RA at *p *< 0.05 were also analyzed in a multiple SNP model.

For the genome-wide data set, single-SNP associations with RA were assessed using a χ^2 ^test when all three genotypes were observed and the least frequent genotype observation was more than five. In situations in which these criteria were not met, Fisher's exact test was used to test the association. The *p*-values were then used to rank the significance of all SNPs.

### Identification of phenotypic subgroups among RA patients

An unsupervised clustering analysis was carried out on the RA cases using the detailed phenotypic data (demographic and disease-related clinical and laboratory variables) to identify clinically distinct subgroups of RA patients. For the purpose of the analysis, the phenotypic variables were dichotomized using a threshold of the 75^th ^percentile for AgeOnset (≥50), TenderJtCt (≥13), SwollenJtCt (≥13), JAMScore (≥52), SeverityLH (≥5), and SeverityRH (≥5). DRB1_1 and DRB1_2 marker variables were combined and dichotomized on the basis of the presence or absence of RA-associated epitopes (*01 and *04 alleles). Patient self-reported American Rheumatism Association (ARA) sub-scores and the overall ARA score were not included in the analysis. All other clinical measurements, except BMI (51% missing), were used in the analysis.

Clustering analysis was carried out using modified 'Jaccard' coefficient (proportion of non-zero pairs that are similar between subjects) as the distance measure [6]. Multidimensional scaling (MDS) of the distance matrix was used to visualize and identify sub-groups in the patient population. After identifying subgroups of RA patients characterized by clinical and laboratory phenotypes, we further investigated whether the SNPs were associated with the sub-groups. Specifically, we attempted to predict the different subgroups within RA cases using the SNP data from the candidate gene study.

### RA classification using RFs

In addition to the conventional association analysis methods that were used in the study to identify RA susceptibility loci, we also used RFs [3]. The method was used for classification or prediction of cases and controls using the SNP data, and five-fold cross-validations (CVs) were used to evaluate classification accuracy. The receiver operating characteristic (ROC) curve was calculated based on the vectors of sensitivity and specificity for each of the five CVs. The values of area under curve (AUC) from the five CVs were averaged to compare the predictive ability and stability.

All of the 17 candidate gene SNPs and the 5742 SNPs from the genome-wide linkage panel were included in the RFs model. RFs were also applied using selected subsets of SNPs as predictor variables. These subsets were selected on the basis of their ranking on the importance measurement implemented in the RFs package. The SNPs that were highly ranked were selected for the RFs model.

All statistical analyses were performed using the R statistical software package (version 2.1.0) from R Project . The R package for Random Forests (randomForest 4.5.-15) was installed and utilized following the instructions.

## Results

### Candidate gene analysis of RA status

The SNPs associated with RA status at *p *< 0.05 are summarized in Table 1. The PTPN22*rs2476601 and SUM04*rs237025 loci showed the strongest association to RA among the significant ones. In the multivariate model, which included all five associated SNPs, *PTPN22 *and *CTLA4 *were found to be independently associated (*p *< 0.05) with RA with a modest effect.

**Table 1:** Summary of significant associations (*p *< 0.05) between RA and candidate genes

	Single-gene model		Multiple-gene model	
		
Gene*SNP	Carrier-test	Genotype-test	*p*-Value	OR (95% CI)
PTPN22*rs2476601	<0.0001	<0.0001	<0.0001	2.43 (1.82, 3.24)
CTLA4*CT60	0.0060	0.0172	0.0115	0.72 (0.56, 0.93)
HAVCR1*5509_5511delCAA	0.0339	0.0640	0.0731	0.80 (0.63, 1.02)
SUM04*rs237025	<0.0001	0.0002	0.1044	1.39 (0.94, 2.04)
MAP3K71P2*rs577001	0.0012	0.0017	0.4527	1.14 (0.80, 1.63)

Using all of the 17 candidate gene SNPs, the predictive ability of the RFs model was estimated by the ROC curve with five-fold cross validation. The low average AUCs of data set 1 and 2 (0.59 and 0.54) indicated poor predictive ability in both data sets. This did not improve by using the three most important SNPs ranked by the RFs model (AUCs = 0.59 for both data sets). Furthermore, SNPs PTPN22*rs2476601 and SUM04*rs237025 are consistently ranked the top two on the variable importance score in both data sets. Interestingly, both SNPs show strong associations in the univariate GEE analysis (Table 1).

### Candidate gene analysis of subgroups among RA patients

We investigated whether there are phenotypically distinct subgroups among RA patients. Such subgroups may represent etiologically or genetically distinct entities among RA patients, and therefore consideration of these subgroups in a genetic association study may lead to an increase in the power to identify susceptibility genes.

**Figure 1:**
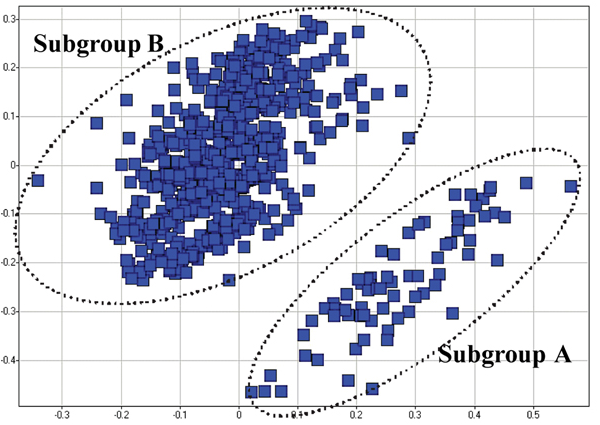
**Two distinct clusters of RA patients in a multidimensional scaling (MDS) plot**. The X-axis and the Y-axis represent the dimensions with the two largest eigenvalues generated by the MDS algorithm.

Figure 1 shows the separation of the two clusters, for which a cluster validity algorithm was employed to determine statistical significance. The empirical distribution of a cluster validity statistic, within-to-between ratio, was computed from 10,000 permutations of the partition vector. The test statistic was defined as the ratio of average within cluster distances to the average between cluster distances for a partition vector. The clusters (subgroups A and B) shown in Figure 1, was calculated to be significant at *p*-value 0.01. These phenotypic clusters are correlated with a set of clinical measures, such as ARA criteria score [7], which reflect the disease severity.

Using the regression analysis method, we did not identify significant associations between SNPs and cluster status among RA patients, or additional SNPs when only severe cases and controls were analyzed. The RFs model predicted the two distinct clusters among RA cases with an overall predictive ability of 0.56 as measured by the AUC of the ROC curve (Table 2). In addition, we excluded the "mild" group (subgroup A in Figure 1) in order to sort out the "severe" group (subgroup B in Figure 1) from the controls. The RFs model was able to predict the two groups with an overall predictive ability of 0.62 (Table 2). The three most important SNPs for the prediction were PTPN22*rs2476601, MAP3K71P2*rs577001, and RUNX1*rs2268277. RUNX1*rs2268277 was not identified in our association analysis and our previous classification, and therefore could be an additional putative association.

**Table 2:** Summary of classification accuracy rates for different classification schemes

RFs classification	Sensitivity	Specificity	AUC of ROC
All cases vs. Controls	57. 5%	60.6%	0.59
Cluster A vs. Cluster B	77.1%	36.3%	0.56
Cluster B vs. Controls	67.1%	52.3%	0.62

### RFs analysis using genome-wide linkage SNP panel

**Figure 2:**
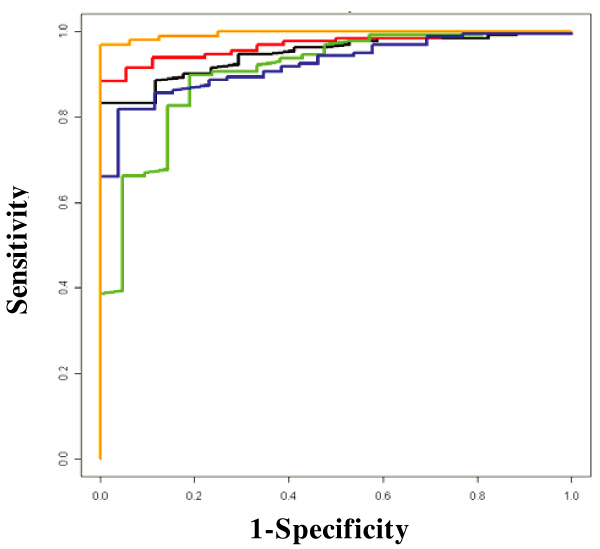
**ROC curve of five-fold CV using RFs with the 500 most important SNPs**. For each CV, a prediction model is built by using the training dataset and the ROC curve is generated by comparing the predicted RA status with the true RA status in the testing dataset. Each color curve represents prediction accuracy of one of the five CVs.

**Figure 3:**
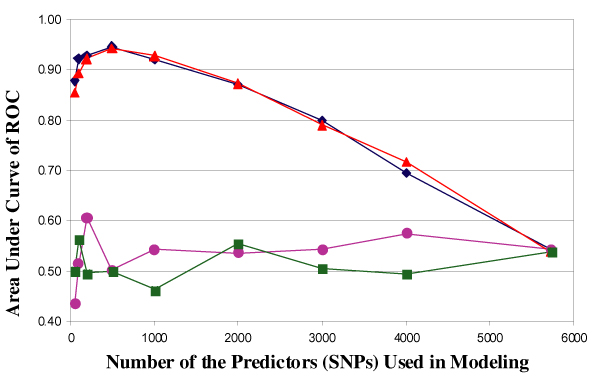
**Reducing dimensionality can improve the predictive ability of RFs**. , Using the most important predictors in data set 1; , using the most important predictors in data set 2; , using random predictors in data set 1; , using random predictors in data set 2.

We examined the predictive ability of RFs using the 5742 genome-wide linkage SNPs. Using the classic training-testing strategy with five-fold CV and the AUC of the ROC curves, we found that the RFs model did not build a reliable prediction model for RA status in either data set 1 or 2. However, when we only used SNPs that were highly ranked on the importance score measure (e.g., the top 500 SNPs), high sensitivities and specificities were consistently obtained in all five CVs for both data set 1 (Figure 2) and 2 (similar results, not shown). The sensitivities and specificities were approximately 90% and 80%, respectively. Figure 3 shows the impact of the number of selected SNP predictors. Using the AUC of the ROC curve to present the predictive ability of each RFs model, we found that the overall predictive ability declined when more than the 500 top ranking SNPs were used to build the model. When compared to the randomly selected SNPs in RF modeling, we concluded that the predictive ability can be significantly improved by selecting the most important SNPs and the improvement was not due to the random effects (Figure 3). When we estimated the most important SNPs only in the training data set for each CV, the improvement of predictive ability was not as obvious as in the full data set. This suggests that the size of the data set may not be sufficient and each training set could not represent the whole data set. Therefore, the best set of predictors identified from each training set would not generalize well across the whole data set.

We generated two replicate data sets in this study to compare the replicability of variable selection using the RFs method and the regular SNP association test. In Table 3, the SNPs identified in both data sets are summarized. In general, among top ranked SNPs by either Random Forests or the association test, the numbers of shared SNPs between the two data sets were not significantly greater than those expected by chance. Only the top 200 SNPs estimated by RFs had a significant overlap (*p*-value was calculated by comparing to the profile of shared SNP numbers which was generated by 10,000 random draws from 5742 SNPs), albeit a small number. There are a number of reasons for the lack of replicability in variable selection. For example, the limited sample size, the weakness of individual SNP effects, the insufficient SNP coverage in the genome, the sampling strategy itself, and over-fitting can all contribute to the inconsistency across samples.

**Table 3:** The overlap of top SNPs using RFs or association test from both data sets

	RFs		Association test	
		
The most significant SNPs from both data sets	Number of common SNPs	*p*-Values	Number of common SNPs	*p*-Values
Top 50	0	NA	1	0.357
Top 100	2	0.525	2	0.525
Top 200	12	0.046*	8	0.405
Top 500	49	0.205	48	0.265
Top 1000	191	0.068	191	0.068
Top 2000	679	0.865	720	0.113
Top 3000	1588	0.216	1576	0.413
Top 4000	2820	0.058	2815	0.103

## Conclusion

As an example of application, we used RFs for the analysis of the NARAC data sets and found that the candidate gene SNPs did not predict RA patients and control subjects accurately. This is despite including SNPs (either as a subset or together with the other candidate gene SNPs) that have been found to be associated in multiple studies [4,8]. However, using the top 500 SNPs ranked by the importance score from the genome-wide linkage panel of 5742 SNPs, we were able to accurately predict RA patients and normal subjects with sensitivity of approximately 90% and specificity of approximately 80%, which was confirmed by five-fold cross-validation and replication across the two data sets.

The RFs algorithm is known to be more resistant to "over-fitting" and the noise variables [3] than conventional regression analysis. In this study, we demonstrated that the analysis strategy needs to be carefully designed for high-dimensional analysis. For instance, by using only the 500 highest ranked SNPs (out of the 5742 SNPs) in the RFs model, the predictive ability was greatly improved compared to using all SNPs. This indicates that RFs may not be resistant to too many "noisy" predictors such as the 100,000s of genome-wide SNP markers. For a specific disease or disease-related trait, only a small portion of all SNPs might be relevant. Therefore, the signal-to-noise ratio has to be optimized by reducing the SNP list to feed into the classification model.

Although we have developed a strategy to generate two replicate samples that were independent within single samples but related across the samples, the genetic effects were not replicated by comparing the most important SNPs in either RFs or by univariate SNP association tests using the genome-wide SNPs. Such lack of replicability in predictive SNPs suggests that the genetic effects may not be robust when the effect size of each SNP is minimal and/or the environmental factors or other covariates were not taken into account in the model. Therefore, in analyzing high dimensional genomic data, it may be essential to incorporate the non-genetic factors that contribute to the risk of the disease. In the analyses of predicting common disease status using the rich genome-wide SNP data, we believe the machine learning methods such as RFs can play an important role in understanding the complicated genetic structures. In addition, further investigations are needed to optimize the algorithms of the RF approach as well as to understand the limitations in attempting to achieve reliable predictive models.

## Competing interests

The author(s) declare that they have no competing interests.
